# Commercially Accessible High-Performance Aluminum-Air Battery Cathodes through Electrodeposition of Mn and Ni Species on Fuel Cell Cathodes

**DOI:** 10.3390/mi14101930

**Published:** 2023-10-14

**Authors:** Paloma Almodóvar, Belén Sotillo, David Giraldo, Joaquín Chacón, Inmaculada Álvarez-Serrano, María Luisa López

**Affiliations:** 1 Albufera Energy Storage, 28001 Madrid, Spain; dgiraldo@ucm.es (D.G.); joaquin.chacon@albufera-energystorage.com (J.C.); 2 Departamento de Física de Materiales, Facultad de Física, Universidad Complutense de Madrid, 28040 Madrid, Spain; bsotillo@ucm.es; 3 Departamento de Química Inorgánica, Facultad de Química, Universidad Complutense de Madrid, 28040 Madrid, Spain; ias@ucm.es (I.Á.-S.); marisal@ucm.es (M.L.L.)

**Keywords:** metal-air batteries, Al-air, electrodeposition

## Abstract

This study presents a cost-effective method for producing high-performance cathodes for aluminum-air batteries. Commercial fuel cell cathodes are modified through electrodeposition of nickel and manganese species. The optimal conditions for electrodeposition are determined using a combination of structural (Raman, SEM, TEM) and electrochemical (LSV, EI, discharge curves) characterization techniques. The structural analysis confirms successful incorporation of nickel and manganese species onto the cathode surface. Electrochemical tests demonstrate enhanced electrochemical activity compared to unmodified cathodes. By combining the favorable properties of electrodeposited manganese species with nickel species, a high-performance cathode is obtained. The developed cathode exhibits capacities of 50 mA h cm^−2^ in aluminum-air batteries across a wide range of current densities. The electrodeposition method proves effective in improving electrochemical performance. A key advantage of this method is its simplicity and cost-effectiveness. The use of commercially available materials and well-established electrodeposition techniques allows for easy scalability and commercialization. This makes it a viable option for large-scale production of high-performance cathodes for the next-generation energy storage devices.

## 1. Introduction

Metal-air batteries such as Li-air, Zn-air, Mg-air, and Al-air batteries are promising for the future generation of energy for mobility and stationary applications because they use oxygen from the air as one of the battery’s main reactants, reducing the weight of the battery and freeing up more space devoted to energy storage. Among all these metal-air batteries, Li-air shows the highest theoretical gravimetric capacity (3860 A h kg^−^^1^). However, this technology poses significant safety problems related to the presence of Li. Al-air technology presents the second highest gravimetric capacity (2980 A h kg^−^^1^), just after Li-air, and the highest volumetric capacity. In addition, these systems do not exhibit many of the environmental and safety problems related to Li based batteries, and their disposable components are fully recyclable [1,2]. 

Metal-air cathodes are composed, usually, of four parts: a current collector (to ensure the good electrical conductivity), a catalyst layer (to ensure the oxygen reduction reaction, ORR), a carbonaceous gas diffusion layer—GDL (responsible of an efficient reduction of oxygen by the contact between oxygen, the electrolyte and the catalyst)—and a O_2_ permeable membrane (to ensure the O_2_ flow, while preventing electrolyte leakage to the outside). There are different methods and patents describing the manufacture and production of these air cathodes. However, in most cases, they describe how a paste/slurry/suspension of the catalytic material is initially added to the GDL carbon by using binders and solvents (which are then removed with high temperature treatments). Afterward, a metallic mesh is placed in the catalyst containing face and a Polytetrafluoroethylene (PTFE) film in the contrary face, and is finally pressed all together in a hot roll press machine [3]. However, despite the advancements in research and development, these methods are often challenging to implement on an industrial scale. Scaling up from laboratory processes to commercial production poses significant difficulties. Additionally, the cost of the components used in metal-air cathodes, such as the catalytic materials and specialized membranes, can also hinder their commercialization. Due to these factors, commercially available metal-air cathodes are currently limited, making it challenging to bring metal-air batteries to the market at a large scale. Researchers and manufacturers are actively working towards developing more scalable and cost-effective production methods to address these challenges and enable widespread adoption of metal-air batteries.

The most widely used catalysts in metal-air batteries are manganese oxides, in particular MnO_2_, since they have a very good relationship in terms of cost and electrochemical activity [4,5]. However, due to their low conductivity, they result in cathodes that present low voltages and low power and need to be doped with different materials, such as cobalt nickel or form composites with carbon to improve these shortcomings [6,7].

On the other hand, nickel hydroxide has been extensively studied in various energy storage systems, such as Ni-Fe and Ni-Cd, or in supercapacitors [8,9]. However, in metal-air batteries, its electrochemical activity has rarely been studied, and it has only been used as a dopant to improve the conductive properties of other materials or as a component of the current collector in different systems [10,11].

Specifically, aluminum-air batteries consist of an air cathode, an aqueous electrolyte, and an aluminum anode. Within aqueous electrolytes, there are two dominant lines of research, alkaline electrolytes based on KOH or NaOH solutions and neutral electrolytes based on NaCl. The main problem with alkaline electrolytes is the spontaneous self-corrosion of the aluminum anode that occurs when it makes contact with them, which greatly limits the lifetime of the battery. In the case of neutral electrolytes, self-corrosion of the aluminum is avoided, and a passivation layer appears on the aluminum surface which reduces the reactivity of the anode and generates parasitic reactions during the electrochemical activity of the battery [1,12]. Different ways of suppressing these effects have been studied, such as the search for commercial anti-corrosive alloys or the coating of the aluminum anodes with a thin layer of carbon that avoids the passivation of the anode when it meets the electrolyte [13]. The right combination of these can avoid these problems and lead to promising energy storage systems. This work presents a novel method that offers a quick, simple, and low-cost approach to transform commercially available fuel cell cathodes into high-performance cathodes for aluminum-air batteries. The transformation involves the electrodeposition of catalytically active nickel and manganese species onto the surface of the cathodes. By utilizing this method, it becomes possible to compare and investigate the electrochemical activity of various electrodes based on nickel and manganese oxides in primary aluminum-air batteries with a neutral electrolyte.

The results obtained from this study provide valuable insights into the combination of nickel and manganese oxides as an electrode with exceptional electrochemical properties in aluminum-air batteries. Moreover, this approach offers the advantage of using commercially available materials and well-established electrodeposition techniques. By employing this simple method, it becomes feasible to produce cathodes for metal-air batteries that can be easily commercialized. Furthermore, the findings from this work have the potential to be applied to other batteries within the metal-air family, thereby broadening the scope of its practical applications.

## 2. Materials and Methods

### 2.1. Cathode Synthesis

A 2M solution of the corresponding metal nitrate in each case (Mn(NO_3_)_2_·4H_2_O or Ni(NO_3_)_2_·6H_2_O, both from Sigma–Aldrich, St. Louis, MO, USA) was prepared in 100 mL distilled water. Moreover, 2M solutions of 9:1, 7:3, 3:2, and 1:1 of Ni:Mn ratio were also prepared. A glassy carbon electrode—positive electrode—and different commercial fuel cell electrodes (Freudenberg H23I2)—negative electrode—of 2.5 × 2.5 cm (surface immersed in the solution) were placed in each solution at a constant distance. Between the electrodes, currents between 20 and 100 mA and varying times between 1 and 10 min were applied with an 8-channel Arbin Instruments BT2143 workstation (Munich, Germany) to obtain the electrodeposition of different manganese and nickel species as a result. Once the electrodeposition was completed, the cathodes were dried at 40 °C for 10 min. It is worth noting that despite the different electrodeposition conditions applied to the samples, the variation in the amount of electrodeposited material between them was practically negligible, ranging from 10 to 13 mg in all cases. This is why all the electrochemical results obtained from these samples would be expressed in cm^−^^2^.

### 2.2. Characterization

Scanning electron microscopy (SEM) images were obtained in a FEI Inspect-S SEM instrument. Energy dispersive X-ray microanalysis (EDS) elemental mappings were acquired with a QUANTAX 70 detector (Bruker, Berlin, Germany) attached to a Hitachi TM3000 SEM microscope working at 15 kV. Micro-Raman measurements were carried out at room temperature in a Horiba Jobin-Ybon LabRAM HR800 on an Olympus BX 41 confocal microscope system with a 633 nm He-Ne laser. High-resolution transmission electron microscopy (HRTEM) images, electron diffraction (ED) patterns, EDS spectra, and mapping in STEM mode were acquired in a JEOL 300FEG electron microscope (Nieuw-Vennep, The Netherlands). The samples were prepared by crushing the powders under n-butanol and dispersing them over copper grids covered with a holey carbon film.

### 2.3. Electrochemical Characterization

To perform the electrochemical characterization, a laboratory-scale metal-air cell prototype was designed with a 2 × 2 cm^2^ open window. Different electrodeposited cathodes, a 2M NaCl solution-based electrolyte, and a 2 × 2 cm^2^ commercial 7475 aluminum anode coated with a 0.025 mm layer of carbon black (Cabot) and PVDF (Sigma–Aldrich, St. Louis, MO, USA) in a 3:2 ratio were placed on it. E-type glass fibber was placed on top of the aluminum, which was utilized as a separator to avoid a short-circuit between the anode and cathode. The electrochemical tests were carried out using an 8-channel Arbin Instruments BT2143 workstation at room temperature. Once the cell was assembled, it was left for 1 h to allow for good impregnation of the electrodes, and the open-circuit voltage (OCV) was recorded and the possible self-discharge of the cell was observed. The cut-off potential for all cases was 0.0 V. Constant current density discharges of 5 mA/cm^2^ were applied to explore the evolution of the voltage. Discharging times and specific capacities referred to the 2 × 2 cm^2^ open window. Linear sweep voltammetry (LSV) curves were performed on the same device and in the same voltage range at a rate of 1 mV/s. 

Electrochemical impedance spectroscopy (EIS) data were obtained by applying an AC voltage of 5 mV in the (0.01–100 kHz) frequency range by using a battery tester Biologic BCS—810.

All measurements were repeated at least three times to ensure reproducibility.

## 3. Results and Discussion

Initially, different commercial cathodes were electrodeposited with nickel and manganese nitrate solutions as described in the Materials and Methods section. We can distinguish different electrodeposition times and currents for the two solutions as detailed in Table 1.

**Figure 1:**
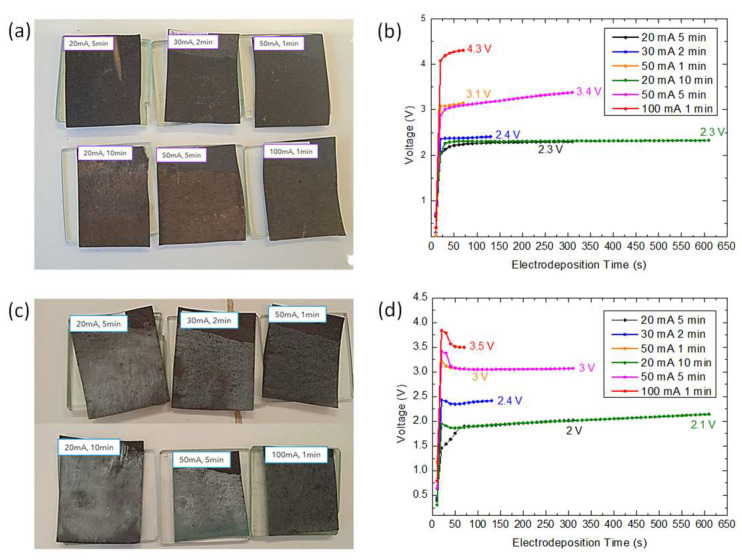
Images of the fuel cell cathodes electrodeposited at different currents and times in (**a**) a manganese nitrate solution with corresponding (**b**) V vs. t electrodeposition curves, and (**c**) a nickel nitrate solution with corresponding (**d**) V vs. t electrodeposition curves.

The different electrodeposited cathodes can be observed in Figure 1. In Figure 1a, we find the cathodes electrodeposited with the manganese nitrate solution, where a layer of slightly brown color uniformly covers the commercial fuel cell cathode, and are the cathodes that have been subjected to longer electrodeposition times and present a more intense color. Figure 1b shows the evolution of the voltage during the electrodeposition time. In all cases, it can be clearly observed how it increases above 2 V, even reaching values higher than 3 V when we work in the range of higher currents. The same occurs with the cathodes electrodeposited with the nickel solution (Figure 1c), which, in this case, show the same layer but with a pale green color (characteristic color in the electrodeposition of nickel nitrate [14]) and again it can be seen that, in all cases, values higher than 2 V are reached in electrodeposition (Figure 1d).

**Figure 2:**
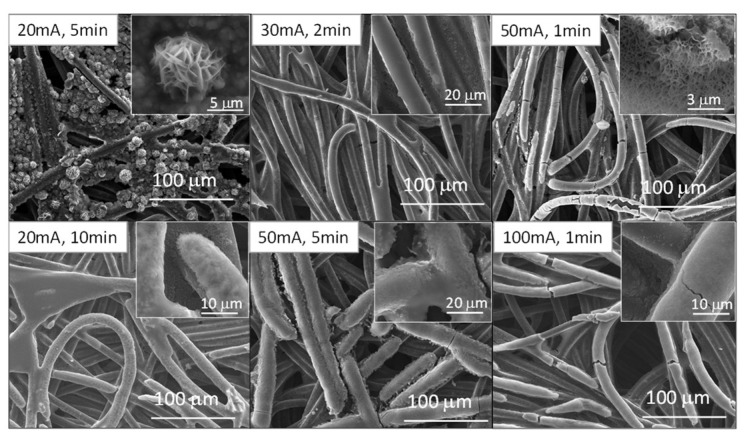
SEM images of commercial fuel cell cathodes electrodeposited in a manganese nitrate solution at different currents and times. Insets show high magnification images of the selected samples.

**Figure 3:**
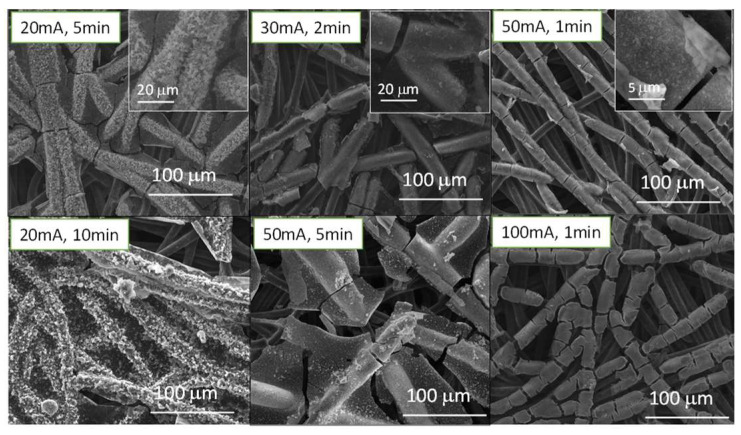
SEM images of commercial fuel cell cathodes electrodeposited in a nickel nitrate solution at different current densities and times. Insets show high magnification images of the selected samples.

If we now study the morphology of the structures deposited on the surface of the cathodes via SEM (Figure 2 and Figure 3), we can observe that the applied current and time are clearly key factors to be considered when it comes to the electrodeposition of these species. In Figure S1, SEM-EDS images of the fuel cell before being electrodeposited can be observed. It is visible that only PTFE-coated carbon fibers are present in the fuel cell and that no other coating or element signal was detected.

Manganese nitrate electrodeposited microstructures can be seen in Figure 2. In the case of the sample electrodeposited at 20 mA for 5 min, the carbon filaments are coated with small platelet-shaped particles that seem to tend to coalesce, along with circular structures with many cavities, similarly to corals. If we increase the current and/or time, we can observe how the small particles disappear and start to form more and more homogeneous coatings on the GDL carbon filaments. In turn, the coral-like structures disappear and seem to merge with this coating, as in the case of the samples subjected to 50 mA for 1 min and 20 mA for 10 min. It can be seen how in the case of the sample subjected to 100 mA for 1 min, the coating of the filaments begins to present a very thick appearance and begins to plug the cavities that allow the circulation of O_2_. Therefore, this sample was discarded to analyze its electrochemical behavior in aluminum-air batteries, and it was determined that the maximum current applied could not exceed 50 mA to obtain satisfactory coatings in this type of configuration.

For samples electrodeposited in the nickel nitrate solution (Figure 3), all the samples present similar morphologies, i.e., uniform coatings are observed on the carbon filaments. At low currents (20 mA), these coatings seem to present a more granular appearance, which tends to disappear and become more fused on the surface as the current is increased (>30 mA). Again, at 100 mA, the coating on the filaments becomes very thick and begins to form plates that merge and may plug the O_2_ penetration holes. For a better comparison with the manganese samples and due to the observed increase trend in the fusing of the coatings, this sample was also discarded from the process to continue the electrochemical study.

**Figure 4:**
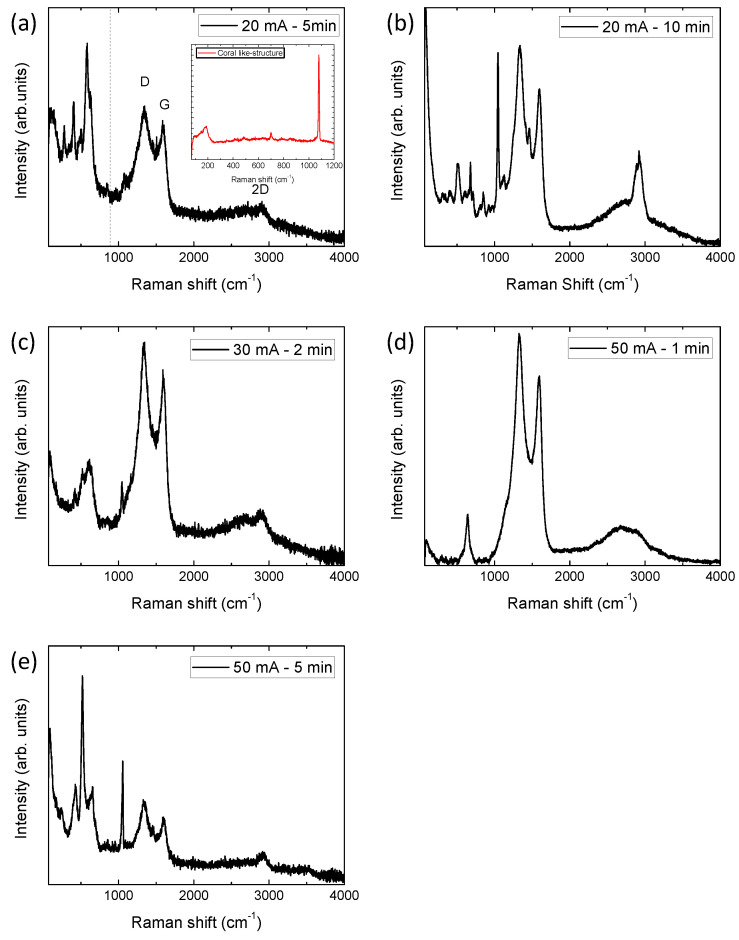
Raman spectra of the different fuel cell electrodeposited in the manganese nitrate solution: (**a**) 20 mA 5 min (inset corresponds to the Raman spectra of the coral-like structures), (**b**) 20 mA, 10 min; (**c**) 30 mA, 2 min; (**d**) 50 mA, 1 min; and (**e**) 50 mA, 5 min.

To determine which phases have been electrodeposited on the carbon fibers, Raman micro-spectroscopy was used. Raman spectra of the samples electrodeposited in the manganese nitrate solution are shown in Figure 4. Table 2 summarizes the phases observed in the electrodeposition process, which depend on the current and time applied. These two parameters determine the different species with different oxidation states that are electrodeposited. Specifically, at lower currents and time, Mn (III) species are found on the surface of the carbon fibers. Meanwhile, at higher currents, Mn (IV) species start to appear. The species obtained are directly related to the voltage values reached during electrodeposition, and are the samples that reached higher voltages (see Figure 1b), i.e., the highest oxidation states. The presence of these phases has been previously observed in the electrodeposition of manganese nitrate following the subsequent reaction sequence, in which the following disproportionation and hydrolysis processes take place [15]:

2Mn^2+^ → 2Mn^3+^ + 2e^−^ (1)

Mn^3+^ + 2H_2_O → MnOOH + 3H^+^ (2)

MnOOH → MnO_2_ + H^+^ + e^−^ (3)

It should be noted that within the 1100–4000 cm^−1^ range, we can observe the signal of the carbon peaks (G, D, and 2D bands) from the fuel cells carbon fibers, superimposed on the signals originating from various manganese phases. In Figure S2, a reference Raman spectrum of the fuel cell cathode, before being subjected to any electrodeposition process, can be found. If we compare this spectrum with the signals referring to electrodeposited carbon fibers, it can be observed how in some cases the carbon signal has suffered an impact after the electrodeposition process, i.e., carbon fibers are altered. Specifically, the 2D band (2700 cm^−^^1^) shifts to higher frequencies, and, even in some samples, a new band associated with C-H vibrations (2950 cm^−^^1^) appears [16,17,18]. This fact is accentuated in the samples that have been subjected to longer electrodeposition times, as they are the samples that were electrodeposited for 10 min and the ones showing the highest intensity of the mentioned C-H band (Figure 4b). This damage may be due to the oxidation and/or reaction of the carbon on the surface during electrodeposition when exceeding 1.8 V [19]. The appearance of a band at 1100 cm^−^^1^ is another indicator of the modification produced in the carbon fibers. This band is directly related to the presence of amorphous carbons and stretching in the C-C bands [20,21]. It is once again more pronounced in the sample that has been electrodeposited for the longest time (20 mA, 10 min). Therefore, this reaction can also interact with the electrodeposited manganese species. Specifically, by measuring the Raman spectrum on the coral-like spheres (inset Figure 4a) in the electrodeposited sample at 20 mA for 5 min, the spectrum obtained is related to MnCO_3_. In the rest of the samples, it is possible to find bands superimposed on the carbon signals, which may be due to Mn-C vibrations. However, as they are fused with the coatings (as seen in SEM), it is difficult to know their exact nature.

**Table 2:** Raman peak assignments of the manganese species electrodeposited on the commercial fuel cell cathodes.

Sample	Raman Peaks (cm^−1^)	Assignment	Reference
20 mA, 5 min	280 403 504 578 631	Mn_2_O_3_	[22]
	700 1080	MnCO_3_	[23]
20 mA, 10 min	325 408 514 612 681 715	MnO_2_, tunnel structure (3 × 3)	[24]
	1042	NO_3_, adsorbed	[25]
30 mA, 2 min	380 485 558 624 655	MnOOH + MnO_2_, mixed forms	[24,26]
	1046	NO_3_, adsorbed	[25]
50 mA, 1 min	640	Mn_3_O_4_	[26,27]
50 mA, 5 min	430 520 650	MnO_2_, tunnel structure (2 × 2)	[24]
	1080	MnCO_3_	[23]

**Figure 5:**
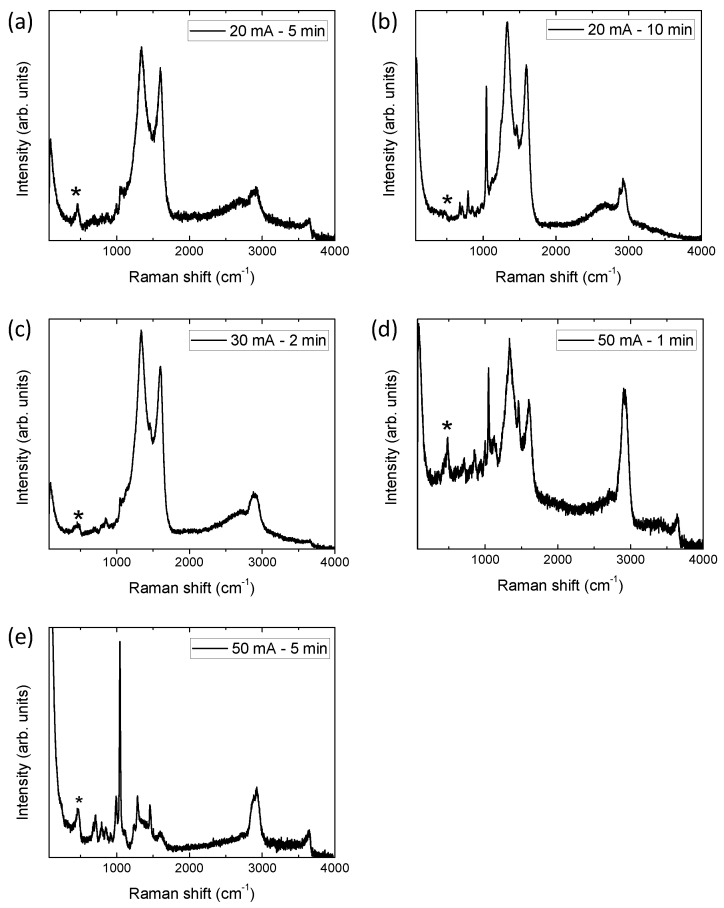
Raman spectra of the different fuel cell electrodeposited in the nickel nitrate solution: (**a**) 20 mA, 5 min; (**b**) 20 mA, 10 min; (**c**) 30 mA, 2 min; (**d**) 50 mA, 1 min; and (**e**) 50 mA, 5 min. Ni(OH)_2_ signal is indicated by *.

In the case of the Raman spectra of samples electrodeposited with nickel hydroxide (Figure 5), we found the presence of Ni(OH)_2_ in all cases. Its presence is confirmed by the broad band centered at ~470 cm^−1^ [8]. The appearance of this phase is expected, since the electrodeposition of nickel from nickel nitrate has been extensively studied and is driven by the following reaction [28,29]:

NO_3_^−^ + H_2_O + 2e^−^ → NO_2_^−^ + 2OH^−^ (4)

The hydroxide ions cause a steep increase in the pH close to the electrode surface and nickel hydroxide precipitation takes place, as follows:

Ni^2+^ + 2OH^−^ → Ni(OH)_2_ (5)

It can be observed that the Ni electrodeposition is more hostile to carbon fibers, since in all cases, even for very low electrodeposition times, a band due to C-H groups (at ~2950 cm^−^^1^) appears. Furthermore, in this case, the current increase has a very significant impact on the carbon fibers, which are the samples electrodeposited at 50 mA and show are the most affected by D, G, and 2D band signals (widening, displacement, and decrease of its signal). This is also related to the observation of a high concentration of H^+^ generated around the glassy carbon during the nickel deposition. On the one hand, this effect allows the nickel electrodeposition on the fuel cell electrode to generate a rich OH^−^ environment (nitrate ions (NO_3_^−^) are reduced to produce hydroxide ions (OH^−^), which reacts with Ni^2+^ ions to form metal hydroxides [30]). On the other hand, it produces the partial reaction of nickel with the carbonaceous electrode [31].

The main structural differences between samples are found in the intercalated species inside the nickel hydroxide matrix. It has been previously observed that, due to the laminar nature of Ni(OH)_2_, it has a high affinity towards the anion intercalation in its structure [8,32]. Predominantly, NO_3_^−^ ion intercalation (1050 cm^−^^1^) can be detected; the presence of NO_3_^−^ ions inside its structure is very frequent when the precursor of this phase is nickel nitrate. In addition, carbonate ions are also present inside Ni(OH)_2_, this process is related to the peaks centered at 870 and 1070 cm^−^^1^ [8,33]. These last ions probably owe their origin to the structural damage suffered by the carbon fibers during electrodeposition. It can be clearly observed that the samples subjected to 20 mA for 10 min, 50 mA for 1 min, and 50 mA for 5 min show the highest intensity of the peaks related to species’ intercalation.

To study the electrochemical behavior of the prepared electrodes in aluminum-air batteries, different experiments were carried out, which prepared the cells as described in the Materials and Methods section and kept the dimensions and the amount of electrolyte constant in all of the cells. On the one hand, LSV curves were performed to analyze the electrochemical activity of the samples in these batteries; on the other hand, discharge protocols at a constant current (20 mA, 5 mA cm^−^^2^) were performed to observe which samples present the highest electrochemical activity in these types of batteries. 

**Figure 6:**
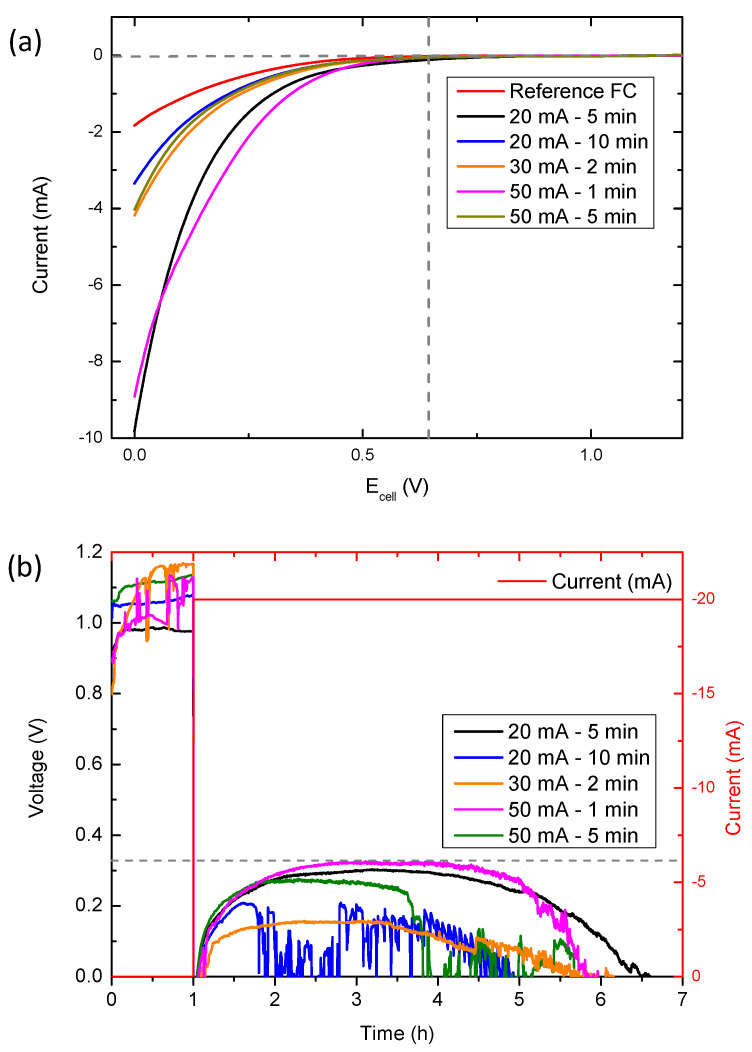
Aluminium-air electrochemical measurements with the electrodeposited manganese nitrate cathodes: (**a**) LSV curves and (**b**) constant current discharge curves.

Based on the LSV curves of the samples electrodeposited in the manganese nitrate solution (Figure 6a), it can be observed how all of the samples start to show activity at a voltage of ~0.6 V, improving, in all cases, the activity of the reference cathode (Reference FC, corresponding to the commercial fuel cell without a catalyst), which does not start showing activity until 0.5 V. By comparing the LSV curves, it can be seen that the samples electrodeposited at 20 mA for 5 min and at 50 mA for 1 min provide the higher ORR kinetic-limiting current densities (>−8 mA, 2 mA cm^−^^2^) [34,35]. This is clearly reflected in the discharge curves (Figure 6b) since they are the samples which provide the highest discharge time, i.e., the highest capacity. In particular, the sample electrodeposited at 20 mA for 5 min stands out, reaching discharge times higher than 5.5 h, which is equivalent to capacities higher than 110 mA h–27.5 mA h cm^−^^2^. This represents an increase of 2 h of discharge compared to the reference fuel cell electrode (Figure S3). A priori, the phases with the best electrochemical activity in this type of battery are the phases with Mn (IV)-derived oxides in their structure [21]. However, in this case, the presence of phases derived from manganese and carbon (MnCO_3_) and the optimal coating of the carbon fibers (thin coatings without blocking the entry of O_2_ into the cell) greatly favor the electrochemical activity of the battery. The presence of Mn-C derivatives has already been observed to considerably improve the activity of primary metal-air batteries compared to pure species [36]. 

Nevertheless, it should be noted that the morphology of the electrodeposited oxides also plays an important role. Both of the samples subject to 20 mA for 5 min and the 50 mA for 1 min show the longest discharge times and we can find the coatings with coral-like cavities in both of them. These morphologies, which are not found or are fused with other structures in the other samples, give rise to a better electrocatalytic activity than that of the samples with MnO_2_ species [21]. This could be due to the presence of thicker and smoother coatings on the carbon fibers in the samples with MnO_2_ species, which may reduce the contact between oxygen, the electrolyte, and the catalyst. To verify this factor, EIS measurements were performed (Figure S4a and Table S1), in which it can be observed how the resistance of the samples increases directly in relation to the thickness of the coatings obtained. With this observation, we can assume that not only the electrocatalytic species that are used are important, but that their morphology and the architecture of the air cathode are also important.

**Figure 7:**
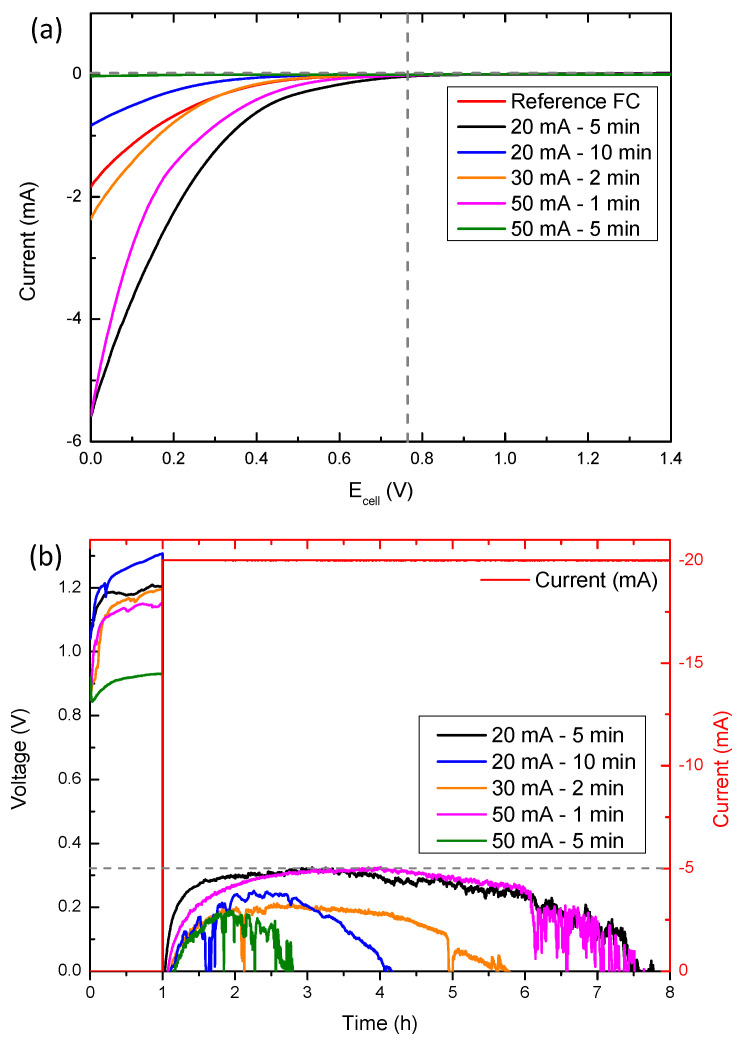
Aluminum-air electrochemical measurements with the electrodeposited nickel nitrate cathodes: (**a**) LSV curves and (**b**) constant current discharge curves.

In the case of nickel electrodeposited samples, the LSV curves (Figure 7a) show that the cathodes electrodeposited at 20 mA for 5 min and 50 mA for 1 min show the highest ORR kinetic-limiting current densities (~−6 mA, −1.5 mA cm^−^^2^). It should be noted that in this case the Ni(OH)_2_, cathodes start their activity at ~0.75 V, significantly increasing the voltage with respect to the reference cathode. Once again, these samples with higher ORR-limiting current densities show the longest discharge time (Figure 7b), reaching 6.5 h (130 mA h–32.5 mA h cm^−^^2^). In particular, the sample electrodeposited at 20 mA for 5 min is the one which shows the most stable discharge curve. These results are also in agreement with those obtained in EIS (Figure S4b and Table S1), which is that the sample electrodeposited with Ni at 20 mA for 5 min offers the lowest resistance. This cathode exhibits an improvement of more than 3 h in the total discharge time with respect to the reference commercial fuel cell cathode.

The results obtained agree with previous observations in relation to the ORR activity of Ni(OH)_2_ with intercalated species [37,38]. The ORR activity of Ni(OH)_2_ increases and stabilizes when it has a certain amount of intercalated species in its structure. However, there comes a point that an increase of these species begins to destabilize its structure and its ORR activity begins to decrease again. Taking this into account, and the results observed in Raman, we can infer that the sample electrodeposited at 20 mA for 5 min presents the optimum number of intercalated species to favor and improve its catalytic activity. On the contrary, the samples that present a higher number of intercalated species, electrodeposited at 20 mA for 10 min and at 50 mA for 5 min, destabilize their structure and considerably reduce their activity.

Comparing the results obtained in different electrodeposited samples, we can conclude that, in both manganese nitrate solution and nickel nitrate solution, the best results are obtained from the electrodepositions performed at 20 mA for 5 min. 

To compare the morphology and structure of these two samples, an additional TEM analysis was performed (Figures S5 and S6). Results corroborate that both show nanometric and porous coatings on the carbon fibers, promoting ORR activity without affecting the contact between oxygen, the electrolyte, and the catalyst. Moreover, from the LSV curves, it can be determined that, while the sample electrodeposited with the manganese species presents a higher ORR kinetic-limiting current density, the sample electrodeposited with nickel presents a higher voltage at which the activity starts. 

**Figure 8:**
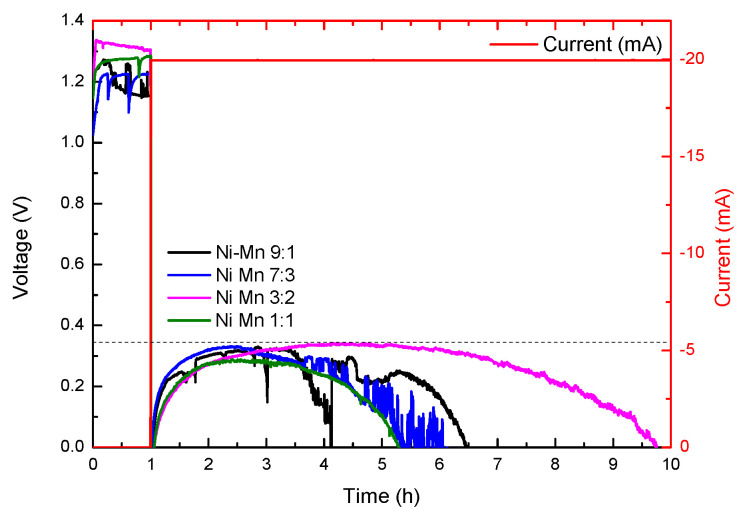
Constant current discharge curves of the different concentrations in electrodeposited Ni:Mn cathodes.

Considering the results obtained above, an attempt was made to combine manganese and nickel ORR activities with the electrodeposition of mixed solutions of nickel and manganese nitrates in different ratios (9:1, 7:3, 3:2, and 1:1 of Ni:Mn, respectively). To evaluate the electrochemical activity of these new combined Ni:Mn cathodes, discharge curves at 20 mA (5 mA cm^−^^2^) with the previously described configuration were again performed. In Figure 8, it can be observed that the sample with a Ni:Mn ratio in a 3:2 solution shows the best electrochemical activity, reaching discharge times of 9 h (180 mA h–45 mA h cm^−^^2^), which corresponds to an increase of more than 100 mA h (25 mA h cm^−^^2^) in the battery capacity with respect to the commercial reference fuel cell cathode. If we again perform LSV (Figure S7) on this electrode, at a Ni:Mn ratio in a 3:2 solution, and compare it with the results previously obtained for the single nickel and manganese electrodeposited electrodes, it can be clearly seen that this electrode combines the good ORR properties of both of them. On the one hand, it maintains the voltage at which the ORR activity begins, which is characteristic of nickel hydroxide. On the other hand, the ORR kinetic-limiting current density reaches similar values to those obtained with the manganese electrode; this proves the efficiency of the combination of the two solutions.

**Figure 9:**
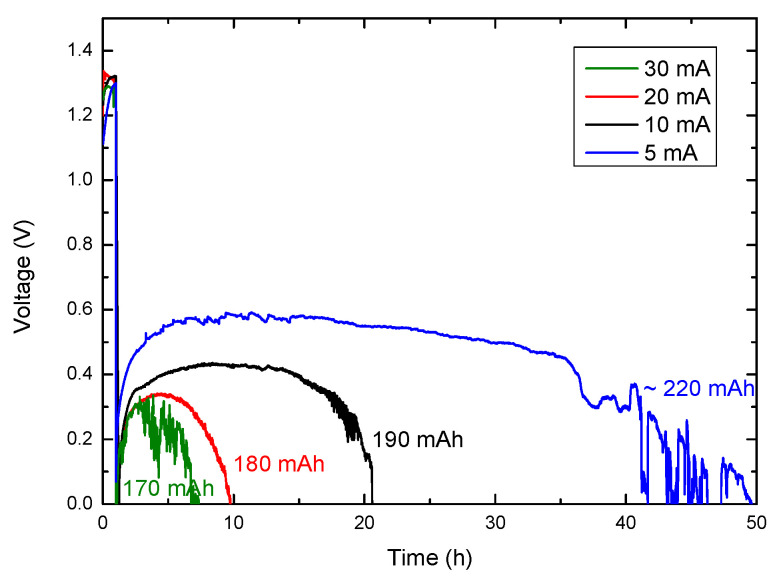
Ni:Mn (3:2) discharge curves at different constant currents: 30 mA (green), 20 mA (red), 10 mA (black), and 5 mA (blue).

In view of the good results obtained for the electrodeposited Ni:Mn (3:2) cathode, new discharges at different currents were carried out to analyze its response (Figure 9). Despite varying the discharge current between 30 and 5 mA (7.5 mA cm^−2^–1.25 mA cm^−2^), we obtain similar capacity values close to 200 mA h (50 mA h cm^−2^). It is evident from these discharge curves that as we increase the discharge current density, the average discharge voltage plateau decreases: ~0.6 V at 1.25 mA cm^−2^, ~0.42 V at 2.5 mA cm^−2^, ~0.33 V at 5 mA cm^−2^, and ~0.24 V at 7.5 mA cm^−2^. However, the power density of the batteries exhibits a logarithmic increase, which is the expected behavior in this type of metal-air battery, appearing to reach its maximum at values close to 1.8 mW cm^−2^ (achieved during a discharge at 30 mA: ~0.24 V at 7.5 mA cm^−2^). These results are depicted in Figure S8. These findings demonstrate how a simple, fast, and inexpensive electrodeposition process can significantly improve the electrochemical activity of commercial cathodes in aluminum-air batteries. 

**Figure 10:**
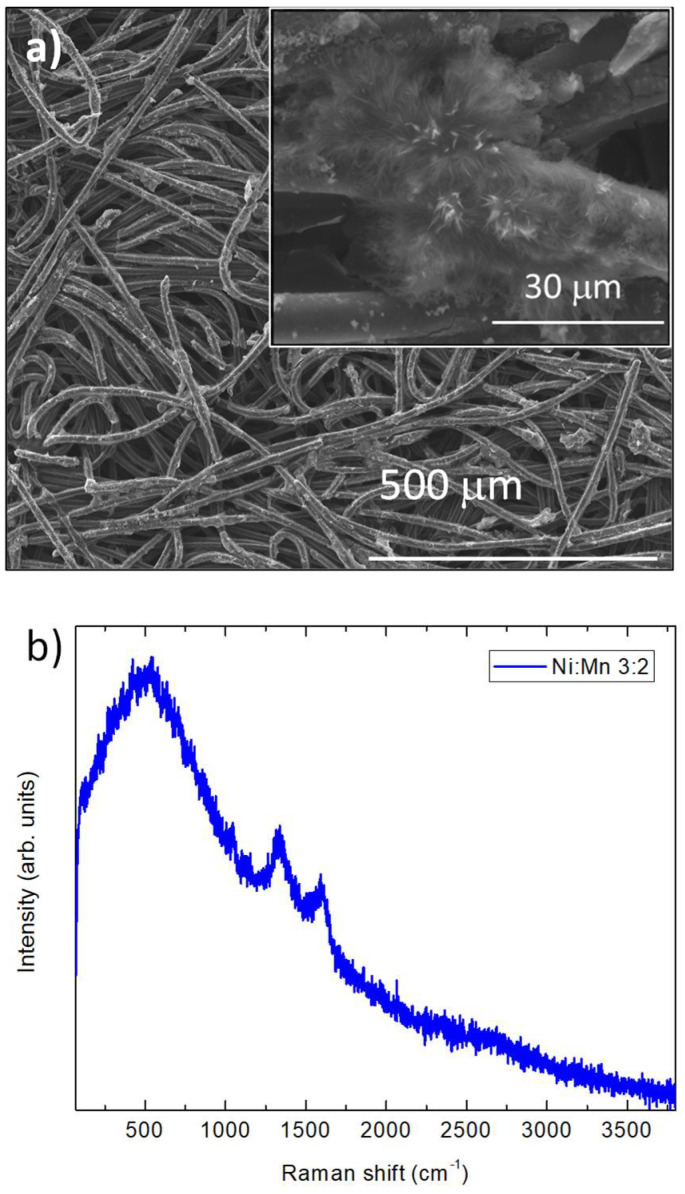
(**a**) SEM images and (**b**) Raman spectra of the Ni:Mn (3:2) electrode.

To analyze this electrode with improved properties in depth, a structural study was conducted. Figure 10a shows the images obtained with SEM, in which a coating with cavities can be appreciated again, reminding us of the coral-like structures which have previously presented a favorable behavior. In the case of the Raman spectrum of this sample (Figure 10b), we can observe the presence of a highly amorphous signal, which is unable to identify any specific peak, and can only identify a broad band in the area corresponding to the characteristic signals of the nickel and manganese oxides and hydroxides [100–1000 cm^−^^1^]. This signal has been observed previously in Ni(OH)_2_ doped with manganese; the presence of manganese could disorder the structure of the nickel hydroxide by intercalating in its layered structure [39].

**Figure 11:**
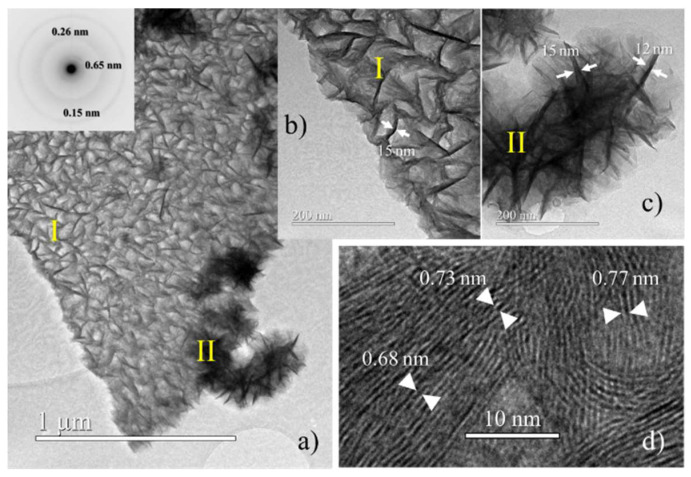
HRTEM images for the Ni:Mn sample at (**a**) low magnification (inset shows the corresponding ED pattern); (**b**,**c**) gathered images corresponding to regions labelled as I and II in image (**a**), respectively; and at (**d**) high magnification.

To understand the origin of this signal, a TEM-EDS study was performed. Figure 11 shows different HRTEM micrographs of the Ni:Mn (3:2) sample. This sample is formed by an interesting nanoarchitecture built from nearly transparent nanosheets (regions I in Figure 11a,b) assembled through stalk-like formations (regions II in Figure 11a,c), which are composed by corrugated nanosheets of about 10–15 nm in width. Both types of morphology (region I and region II) exhibit the same composition of 3Ni:1Mn, as determined from the corresponding EDS spectra (Figure S9). This composition aligns with the initial molar ratios used during electrodeposition and highlights the preference for nickel electrodeposition over manganese. 

The inset of Figure 11a gathers a representative ED pattern, which can be indexed based on the Ni(OH)_2_-type structure, similar to that obtained for the Ni 20 sample electrodeposited at mA for 5 min. As the sample presents very low crystallinity, the periodic contrasts appear only along small distances and it is difficult to determine precise values, as would be the case in well-grown crystals. Thus, the d-spacing values of 0.65 nm could be indexed to the (003) crystal planes of Ni(OH)_2_; however, they are probably more representative of the (003) crystal planes of NiOOH. The other two distances indicated in the ED pattern (inset of Figure 11a), 0.26 and 0.15 nm, could also correspond to both phases. Moreover, the distances corresponding to the (003) crystal planes range between 0.65 and 0.78 nm (Figure 11d), pointing to the probable coexistence of Ni(OH)_2_ and MOOH, with M=Mn, Ni. Furthermore, the EDS mappings (Figure S10) of the Ni:Mn nanosheets indicate compositional homogeneity at a nanoscale. Indeed, Mn doping at the atomic level (up to 6%) has been previously reported as a strategy to induce local contraction of the metal-O/metal bond length, stabilizing the structure in this way [39]. In addition, Mn doping of Ni(OH)_2_ has been proved to promote the production of high-valence Ni^III^OOH [40]. Thus, the stabilization of trivalent Ni and Mn cations should be an interesting electronic feature of the Ni:Mn sample that leads to an improved electrochemical response. 

Lastly, it is important to highlight that all the samples examined in this study were characterized both before and after the electrochemical measurements. These assessments revealed no significant differences in the electrodes, indicating that the cathode elements (the carbon fibers and the electrodeposited catalysts) do not undergo degradation during battery discharge. This suggests their potential for reuse in another battery of the same type, with the sole requirement of having to replace the consumed aluminum. A representative SEM image, along with its corresponding EDS analysis, of the Ni:Mn 3:2 sample after cycling is presented in Figure S11.

## 4. Conclusions

In this work, we have shown that commercial fuel cell cathodes can be converted into high-performance cathodes for metal-air batteries through electrodeposition. Two types of sample series have been tested, one with the electrodeposition of nickel species and another with manganese species. We have determined the optimal parameters for the electrodeposition of both species, which is a current of 20 mA and a deposition time of 5 min. Electrodeposition of manganese species results in high ORR kinetic-limiting current density, while the electrodeposition of nickel presents a higher voltage at which the activity starts. Along with the importance of the electrocatalytic species used, the morphology and the architecture of the air cathode play an important role. A porous coating of the carbon fibers, formed by interconnected sheets or platelet-shaped particles, favors the contact between oxygen, the electrolyte, and the catalyst. The combination of Ni and Mn in optimal proportions produces cathodes with good properties that are merged from both species, leading to the construction of 50 mA h cm^−^^2^ Al-air batteries. In conclusion, this study presents a novel, cost-effective method for producing high-performance cathodes for aluminum-air batteries through electrodeposition of nickel and manganese species. The method in this study offers a readily scalable solution that enhances electrochemical performance. The results highlight its potential for practical applications in emerging energy storage technologies.

## Figures and Tables

**Table 1:** Description of currents and times applied for the different electrodepositions.

Current (mA)	Time (min)
20	5 10
30	2
50	1 5
100	1

## Supplementary Materials

The following supporting information can be downloaded at: https://www.mdpi.com/article/10.3390/mi14101930/s1. Figure S1. SEM-EDS compositional mapping of commercial fuel cell cathode: (a) SE signal, (b) carbon signal and (c) F signal. (d) EDS spectra. Figure S2. Raman spectra of the commercial fuel cell cathode. Figure S3. Constant current discharge curve of the reference commercial fuel cell cathode. Figure S4. EIS Nyquist plots – insets show the equivalent circuits: (a) Manganese nitrate electrodeposited samples and (b) Nickel nitrate electrodeposited samples. Figure S5. HRTEM images for the Mn (20 mA-5min) sample (a) at low magnification (inset shows the corresponding ED pattern indexed based on cubic Mn2O3) and at high magnification in different regions showing the presence of (b) Mn2O3 and (c) MnCO3. Figure S6. HRTEM images for the Ni (20 mA-5min) sample (a) at low magnification (inset shows the corresponding ED pattern indexed based on hydrated Ni(OH)2) and (b) at high magnification. Figure S7. LSV curves comparation between the samples electrodeposited at 20 mA 5 min: Ni:Mn 3:2 (black), Mn 20 mA 5 min (pink) and Ni 20 mA 5min (blue). Figure S8. Discharge voltage and power density vs discharge current density for Ni:Mn 3:2 electrode. Figure S9. EDS obtained from the different HRTEM regions for the Ni:Mn sample. Cu signal originates from the sample holder. Figure S10. (a) STEM image of the Ni:Mn sample and (b) Ni, (c) Mn and (d) O corresponding mappings. Figure S11. SEM image (a) and corresponding EDS (b) of the Ni:Mn 3:2 sample after cycling. Table S1. Relevant results obtained from Nyquist plots fitting.
