# Supplementary material for: Commercially Accessible High-Performance Aluminum-Air Battery Cathodes through Electrodeposition of Mn and Ni Species on Fuel Cell Cathodes
Source: Micromachines (Basel). 2023 Oct 14;14(10):1930. doi: 10.3390/mi14101930 (PMC10609553; doi:10.3390/mi14101930)
Supplement: Supplementary file 1 [file micromachines-14-01930-s001.zip › micromachines-2660068-supplementary.pdf]

Supplementary Material

Commercially Accessible High-Performance Aluminum-Air Battery Cathodes through Electrodeposition of Mn and Ni Species on Fuel Cell Cathodes

Paloma Almodóvar [1,*], Belén Sotillo [2], David Giraldo [1,3], Joaquín Chacón [1], Inmaculada Álvarez-Serrano [3] and María Luisa López [3]

[1] Albufera Energy Storage, 28001 Madrid, Spain; dgiraldo@ucm.es (D.G.); joaquin.chacon@albufera-energystorage.com (J.C.)

[2] Departamento de Física de Materiales, Facultad de Física, Universidad Complutense de Madrid,
28040 Madrid, Spain; bsotillo@ucm.es

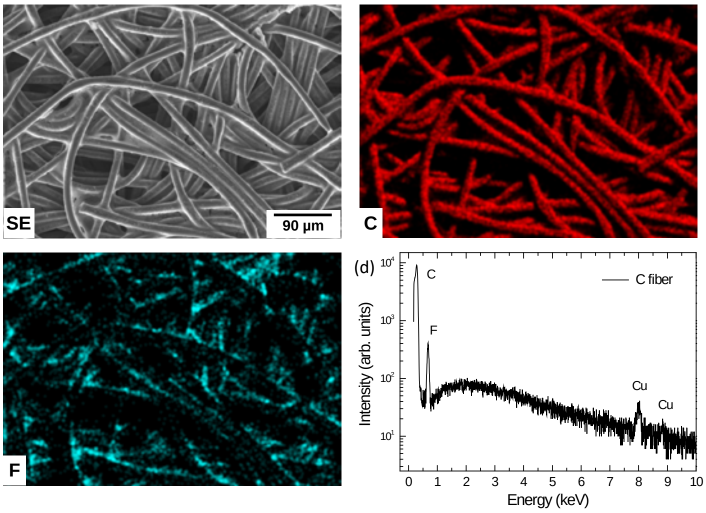

Figure S1. SEM-EDS compositional mapping of commercial fuel cell cathode: (a) SE signal, (b) carbon signal and (c) F signal. (d) EDS spectra.

SEM image of commercial fuel-cell cathode show a dense entanglement of fibers with diameters of about (8-10) μm and lengths in the mm range. SEM-EDS microanalysis show that C is the main element, although F is also clearly detected. The spectrum is represented in log scale in order to visualize elements present in lower concentrations. Cu signal comes from the SEM sample holder. Compositional EDS mappings reveal that F is mainly located at the rim of some fibers as well as in some interstices between them.

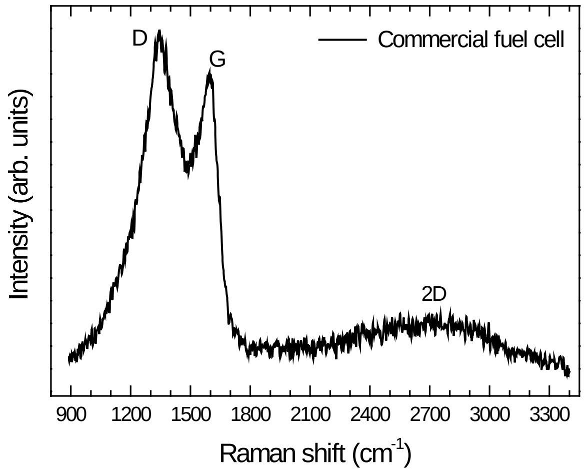

Figure S2. Raman spectra of the commercial fuel cell cathode.

Raman signal from the fibers is quite weak. The corresponding spectrum show two broad bands centered at about 1345 and 1594 cm^{-1} (D and G bands) characteristic of graphitic materials with low crystallinity. No signal is observed in the (80 – 900) cm^{-1} spectral range.

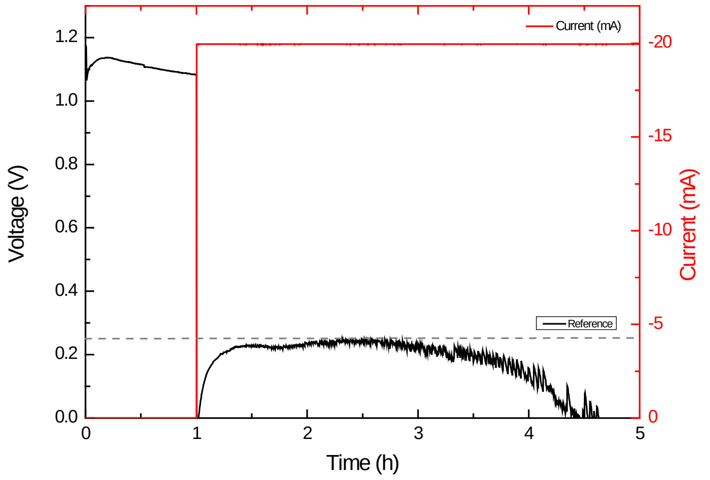

Figure S3. Constant current discharge curve of the reference commercial fuel cell cathode.

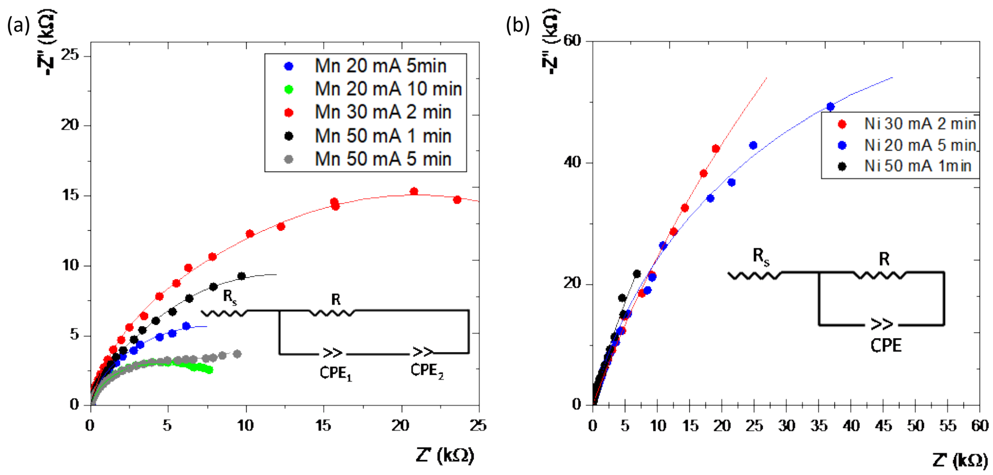

Figure S4.EIS Nyquist plots – insets show the equivalent circuits: (a) Manganese nitrate electrodeposited samples and (b) Nickel nitrate electrodeposited samples.

Table S1. Relevant results obtained from Nyquist plots fitting.

	R_S (Ω)	R (Ω)	C (F. $S^{(N-1)}$)	N
Ni 20 mA 5 min	1.9	$1.48x10^5$	$4.56x10^{-5}$	0.85
Ni 30 mA 2 min	1.9	$4.35x10^5$	$5.83x10^{-5}$	0.79
Ni 50 mA 1 min	1.9	$3.28x10^5$	$1.41x10^{-4}$	0.85
Mn 20 mA 5 min	1.9	$1.26x10^4$	$2.61x10^{-4}$	0.87
Mn 30 mA 2 min	1.9	$4.21x10^4$	$6.06x10^{-4}$	0.79
Mn 50 mA 1 min	1.9	$2.58x10^4$	$1.67x10^{-4}$	0.80

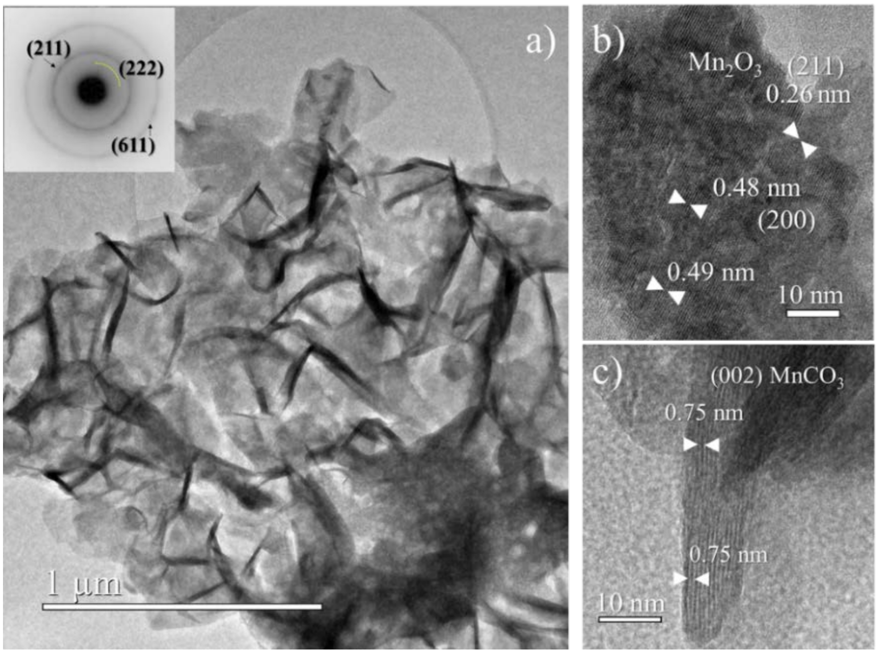

Figure S5. HRTEM images for the Mn (20mA-5min) sample a) at low magnification (inset shows the corresponding ED pattern indexed based on cubic Mn_2O_3) and at high magnification in different regions showing the presence of b) Mn_2O_3 and c) $MnCO_3$.

The inspection for the Mn sample (Figure S 5) reveals the presence of corrugated nanoflakes. Corresponding ED patterns (inset of Figure S 5.a) indicates a very low degree of crystallinity and could be indexed based on Mn_2O_3 (JCPDS no. 00-001-1061). Whereas most of the particle is formed by Mn_2O_3 (Figure S 5.b,) a deeper inspection at higher magnifications reveals the presence of $MnCO_3$ as ribbon-like formations in the outer part of the particles (Figure S 5.c).

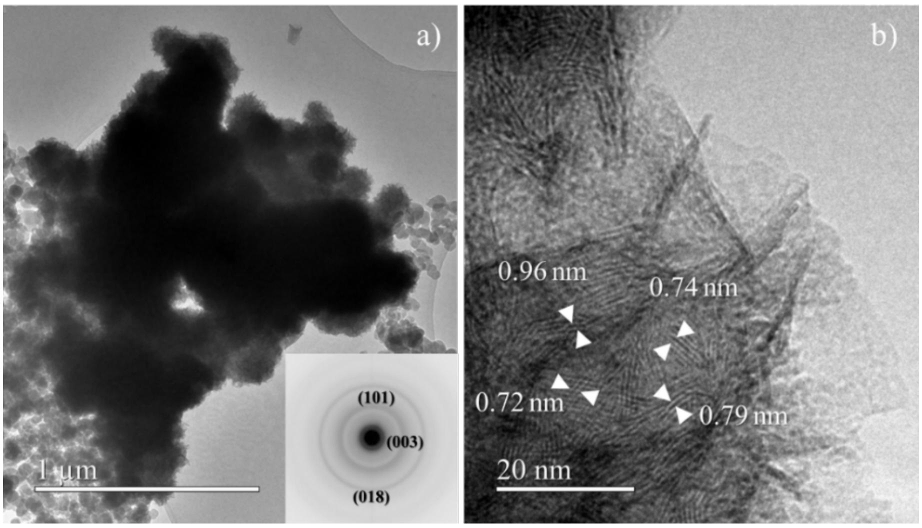

Figure S6. HRTEM images for the Ni (20mA-5min) sample a) at low magnification (inset shows the corresponding ED pattern indexed based on hydrated $Ni(OH)_2$) and b) at high magnification.

In the case of the Ni (20mA-5min) sample, a porous-like architecture built from ultrathin nanosheets is observed to develop over the carbon fibers (Figure S 6.a). The corresponding ED pattern (inset in Figure S 6.a), indexed considering hydrated $Ni(OH)_2$ (JCPDS 38–0715), confirms the very low crystallinity degree of the sample. Thus, *d*-spacing values corresponding to the (003) crystal planes of $Ni(OH)_2$ range between 0.72 and 0.79 nm. Additionally, greater distances up to ~1 nm between contrasts can be appreciated in some regions of the particles. These can be found directly related to the intercalation of species within its structure.

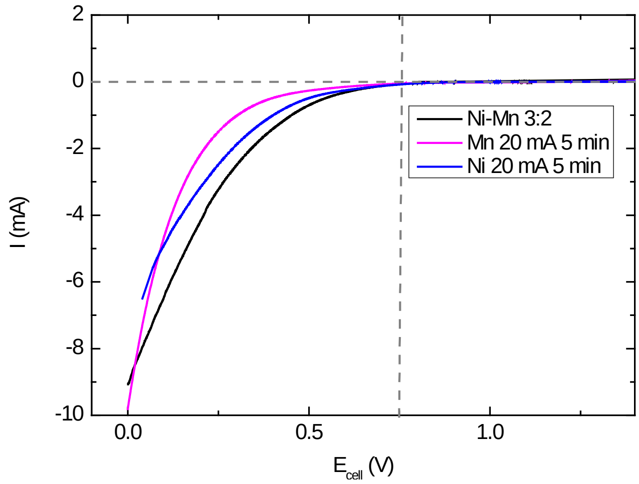

Figure S7. LSV curves comparation between the samples electrodeposited at 20 mA 5 min: Ni:Mn 3:2 (black), Mn 20 mA 5 min (pink) and Ni 20 mA 5min (blue).

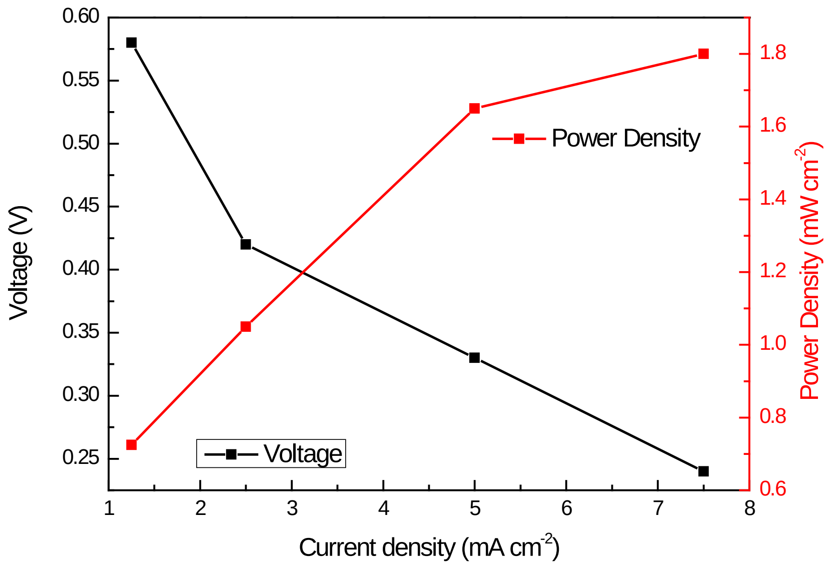

Figure S8. Discharge voltage and power density vs discharge current density for Ni:Mn 3:2 electrode.

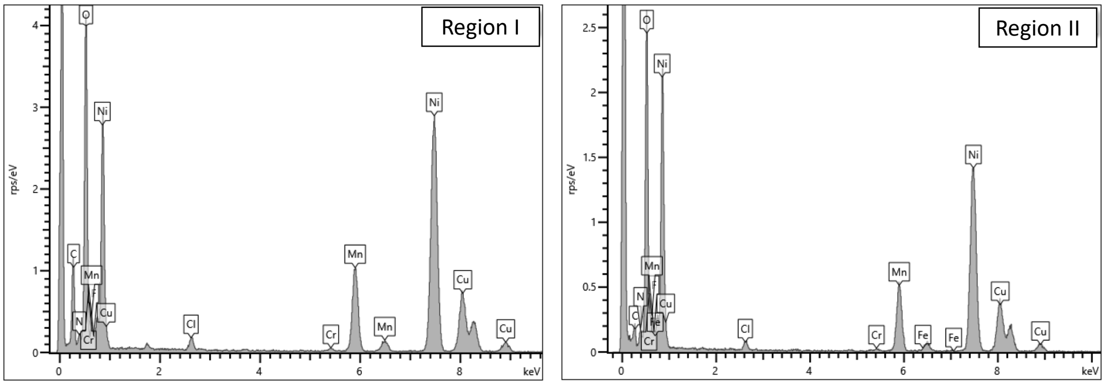

Figure S9. EDS obtained from the different HRTEM regions for the Ni:Mn sample. Cu signal originates from the sample holder.

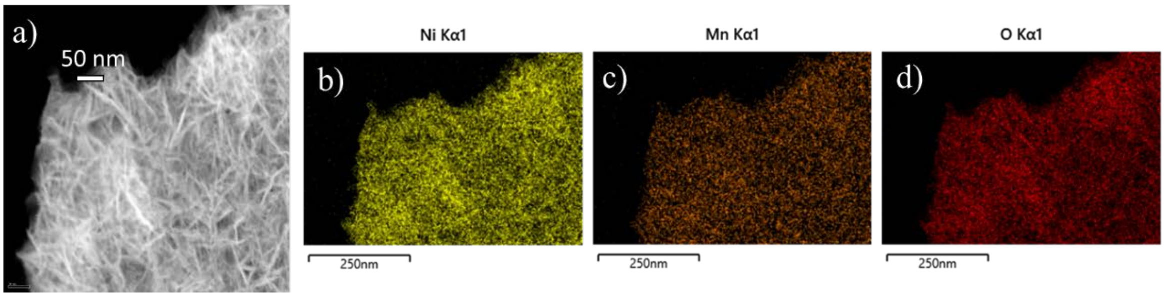

Figure S10. a) STEM image of the Ni:Mn sample and b) Ni, c) Mn and d) O corresponding mappings.

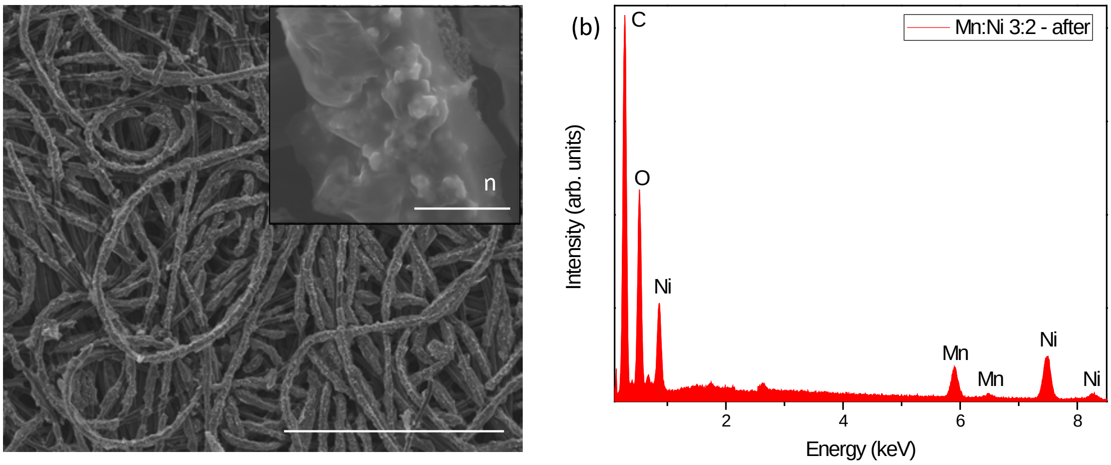

Figure S11. SEM image (a) and corresponding EDS (b) of the Ni:Mn 3:2 sample after cycling.
